# Connectivity and tissue microstructural alterations in right and left temporal lobe epilepsy revealed by diffusion spectrum imaging

**DOI:** 10.1016/j.nicl.2014.07.013

**Published:** 2014-08-01

**Authors:** Alia Lemkaddem, Alessandro Daducci, Nicolas Kunz, François Lazeyras, Margitta Seeck, Jean-Philippe Thiran, Serge Vulliémoz

**Affiliations:** a Ecole Polythechnique Fédéral de Lausanne, Signal Processing Laboratories (LTS5), Lausanne, Switzerland; b Centre d'Imagerie BioMédicale (CIBM-AIT), Ecole Polythechnique Fédéral de Lausanne, Lausanne, Switzerland; c Dpt of Radiology, University Hospital of Geneva, Switzerland; d Epilepsy Unit, Neurology Clinic, University Hospitals and Faculty of Medicine of Geneva, Switzerland; e Dpt of Radiology, University Hospital and University of Lausanne, Switzerland

**Keywords:** Diffusion MRI, Connectome, Tractography, Network measures, DSI, GFA, NODDI, Temporal lobe epilepsy

## Abstract

Focal epilepsy is increasingly recognized as the result of an altered brain network, both on the structural and functional levels and the characterization of these widespread brain alterations is crucial for our understanding of the clinical manifestation of seizure and cognitive deficits as well as for the management of candidates to epilepsy surgery.

Tractography based on Diffusion Tensor Imaging allows non-invasive mapping of white matter tracts in vivo. Recently, diffusion spectrum imaging (DSI), based on an increased number of diffusion directions and intensities, has improved the sensitivity of tractography, notably with respect to the problem of fiber crossing and recent developments allow acquisition times compatible with clinical application.

We used DSI and parcellation of the gray matter in regions of interest to build whole-brain connectivity matrices describing the mutual connections between cortical and subcortical regions in patients with focal epilepsy and healthy controls. In addition, the high angular and radial resolution of DSI allowed us to evaluate also some of the biophysical compartment models, to better understand the cause of the changes in diffusion anisotropy.

Global connectivity, hub architecture and regional connectivity patterns were altered in TLE patients and showed different characteristics in RTLE vs LTLE with stronger abnormalities in RTLE. The microstructural analysis suggested that disturbed axonal density contributed more than fiber orientation to the connectivity changes affecting the temporal lobes whereas fiber orientation changes were more involved in extratemporal lobe changes. Our study provides further structural evidence that RTLE and LTLE are not symmetrical entities and DSI-based imaging could help investigate the microstructural correlate of these imaging abnormalities.

## Highlights

• We performed a whole brain connectivity study in patients with RTLE and LTLE.
• Diffusion Spectrum Imaging (DSI) was used to achieve a better angular resolution.
• DSI was also used to allow investigating the tracts microstructure.
• Temporal changes where more related to the neurite density.
• The extratemporal changes where related to the neurite orientation dispersion.

## 1 Introduction

Patients with temporal lobe epilepsy (TLE) suffer from dysfunctions affecting large-scale brain networks rather than a single focal region (Laufs, 2012). Functional and structural brain imaging studies, as well as invasive electrophysiology studies (Engel et al., 2013; Bernhardt et al., 2013; Van Diessen et al., 2013) have shown that this so-called epileptic network involves temporal and extratemporal regions of both hemisphere (Fahoum et al., 2012). The mapping of these abnormal neuronal networks is an important prerequisite for a better understanding of this condition, particularly when evaluating patients who are candidates for epilepsy surgery.

The development of diffusion-based Magnetic Resonance (MR) imaging and the tractography of white matter fiber tracts have allowed the investigation of the structural alterations underlying these abnormal brain functions (Gross, 2011). MR diffusion-based studies have initially demonstrated local alterations in diffusion measures in the white matter using voxel-based morphometry, however without integration of these abnormalities into identified tracts or networks. This was followed by the mapping of alterations in specific user-defined white matter tracts (limbic circuitry, uncinate fasciculus, arcuate fasciculus) with some correlation to cognitive measures (Aarts et al., 1984; Yogarajah et al., 2008; Concha et al., 2009). Tract-Based Spatial Statistics and similar techniques have revealed widespread bilateral temporal and extratemporal alterations in major white matter tracts (Focke et al., 2008; Voets et al., 2012). Very recently, in an effort to bridge the gap between cortical functional and subcortical structural connectivity studies, a few studies have investigated which cortical regions are affected by these subcortical white matter abnormalities. Using graph analysis applied to structural networks in patients with TLE, they found an altered distribution of connectivity hubs in left TLE vs controls (Liu et al., 2014). In addition, preoperatively increased connectivity in a temporal and extratemporal network was associated to persistence of post-operative seizures (Bonilha et al., 2013).

Fractional Anisotropy (FA) is the measure usually applied to describe white matter tract abnormalities but its microstructural biological correlates remain insufficiently understood. Recent studies have proposed MR-based measures of Intra-cellular Volume Fraction (ICVF) and Orientation Dispersion Index (ODI) as important components of the Fractional Anisotropy (Zhang et al., 2012; Kunz et al., 2014). Diffusion spectrum imaging (DSI) is a recently developed high angular resolution diffusion technique (Wedeen et al., 2005). DSI has been shown to better resolve the ambiguity of fiber crossing encountered in tractography (Cammoun et al., 2012; Granziera et al., 2009) and to provide increased sensitivity towards the detection of short range cortico-cortical connections (Gigandet et al., 2008; Granziera et al., 2012). Due to its multiple b-value properties, DSI also allows estimating finer microstructural properties such as ICVF and ODI to investigate the factors influencing GFA (Generalized Fractional Anisotropy) changes.

Here, we used DSI to estimate the structural connectivity and network properties in right (RTLE) and left TLE (LTLE) patients compared to healthy controls at whole-brain and regional level. We investigated three different structural connectivity matrices, averaging GFA (Tuch, 2004), ICVF and ODI (Zhang et al., 2012) along the tracts. Using a graph theory framework, we then identified cortical regions that significantly contributed to alterations in typical network indices such as the Strength, Efficiency, Shortest Path and Clustering.

## 2 Materials and methods

### 2.1 Patients and controls

22 patients with pharmacoresistant unilateral TLE (13 females and 9 males, age = 33.8 ± 10.1 years, 12 right-sided and 10 left) participated in this study and were recruited from the joint Epilepsy Surgery Program of Geneva and Lausanne, Switzerland. 15 patients had HS (hippocampal sclerosis), 7 patients had no apparent MRI lesion. The clinical diagnosis of TLE was made according to concordant electro-clinico-imaging data by experienced epileptologists (SV, MS). In 9 patients, invasive validation with intracranial EEG and/or seizure-freedom following anterior temporal lobe resection was available. In the remaining HS patients, a concordant non-invasive work-up was obtained. See Table 1 for more detailed information.

In addition, 21 healthy volunteers (8 females and 13 males, age = 31.2 ± 4.8 years) were included. The subjects were acquired on two different sites where a reproducibility study previously showed no scanner bias in the analysis. The two scanners used in the current study are referred to as scanners A and B in Lemkaddem et al. (2012). None of these control subjects had a history of neurological or psychiatric disorders. The ethical committee of two hospitals involved in this work approved this study and a written informed consent was obtained from each participant.

### 2.2 Imaging acquisition

MRI acquisition was performed on a 3 T Trio A Tim System (Siemens, Erlangen, Germany) using a 32-channel head coil. The imaging protocol included a DSI acquisition using a twice refocusing spin-echo with EPI read-out and diffusion gradient scheme minimizing eddy-current induced effects (Reese et al., 2003). The acquisition parameters were: TR/TE = 8500/154 ms; acquisition matrix = 96 × 96; in-plane resolution = 2.2 × 2.2 mm; slice thickness = 3 mm; 44 axial slices; acceleration factor = 2 and partial phase encoding factor = 6/8. It was acquired according to aq4-half protocol, which consists of 128 measurements in a 3D Cartesian grid comprised by the q-space points of a cubic lattice within the hemisphere of 4 lattice units in radius, with a maximum b-value of 6400 s/mm^2^ (Wedeen et al., 2008).

Anatomical images were acquired for cortex parcellation with a T1-weighted MPRAGE (TR/TE = 2300/2.86 ms) and T2-weighted 3D SPACE (TR/TE = 3200/408 ms) with both an acquisition matrix = 256 × 256; voxel size = 1 × 1 × 1.2 mm; 160 sagittal slices; acceleration factor = 2; variable flip angle. The total scanning time was 30 min.

### 2.3 Pre-processing

84 cortical and subcortical regions with anatomical landmarks were mapped from MPRAGE image using Freesurfer 5.0 software (http://surfer.nmr.mgh.harvard.edu). These regions of interest (ROIs) are then co-registered to the diffusion image space using a nonlinear registration tool of FSL (FNIRT) (http://fsl.fmrib.ox.ac.uk).

In order to cope with geometric distortions due to the diffusion Echo Planar Image (EPI) read-out, the T2-weighted image was used for registering the MPRAGE image to the diffusion image. This procedure has the advantage that the T2-weighted image shares the same contrast as the b0 image but is much less distorted. Whole brain tractography was performed in the white matter areas using an in-house streamline-based algorithm adapted to work with DSI data (Daducci et al., 2012; Lemkaddem et al., 2012).

### 2.4 Diffusion model reconstruction and connectivity computation

An ODF (Orientation Distribution Function) was evaluated for a set of vectors representing the vertices of a regular polyhedron, the 362 vertex 6-fold geodesated icosahedron, of mean nearest-neighbor separation = 0.16, rad = 9°. Next, the GFA (Tuch, 2004) that is defined as an analog for q-ball imaging of the FA in DTI was computed from the ODFs. The GFA is expressed as:

$$\mathrm{GFA}=\frac{\mathrm{SD}\left(\mathrm{ODF}\right)}{\mathrm{RMS}\left(\mathrm{ODF}\right)},$$

where *SD*(*ODF*) is the standard deviation of the *ODF* and *RMS*(*ODF*) is its root mean square. Beside the usual indices derived from the ODF such as the GFA, we used a biophysical multi-compartment diffusion model, the Neurite Orientation Dispersion and Density Imaging (NODDI) model (Zhang et al., 2012) to further investigate the different microstructural contributions to the GFA measure. We estimated microstructural white-matter properties by looking at the Intra-cellular Volume Fraction (ICVF) and the dispersion of the fibers that is summarized by the scalar-valued Orientation Dispersion Index (ODI). These new parameters aim to disentangle microstructural contribution of the GFA and are described in detail in Zhang et al. (2012). These parameters have already been applied in other works such as Kunz et al. (2014) and Winston et al. (2014).

Using the NODDI model, a full expression of the normalized signal would be:

$$S=\left(1-\mathrm{ISOF}\right)*\left(\mathrm{ICVF}*\mathrm{Sic}+\left(1-\mathrm{ICVF}\right)*\mathrm{Sec}\right)+\mathrm{ISOF}*\mathrm{Siso},$$

where Sic and ICVF are the normalized signal and volume fraction of the intra-cellular compartment; Sec is the normalized signal of the extra-cellular compartment; and Siso and ISOF are the normalized signal and volume fraction of the CSF compartment.

The ODI is an index providing the degree of dispersion of the fibers by adapting the Watson distribution (Zhang et al., 2012). It ranges from 0 (no dispersion) to 1 (fully dispersed).

### 2.5 Connectivity analysis

**Fig. 1:**
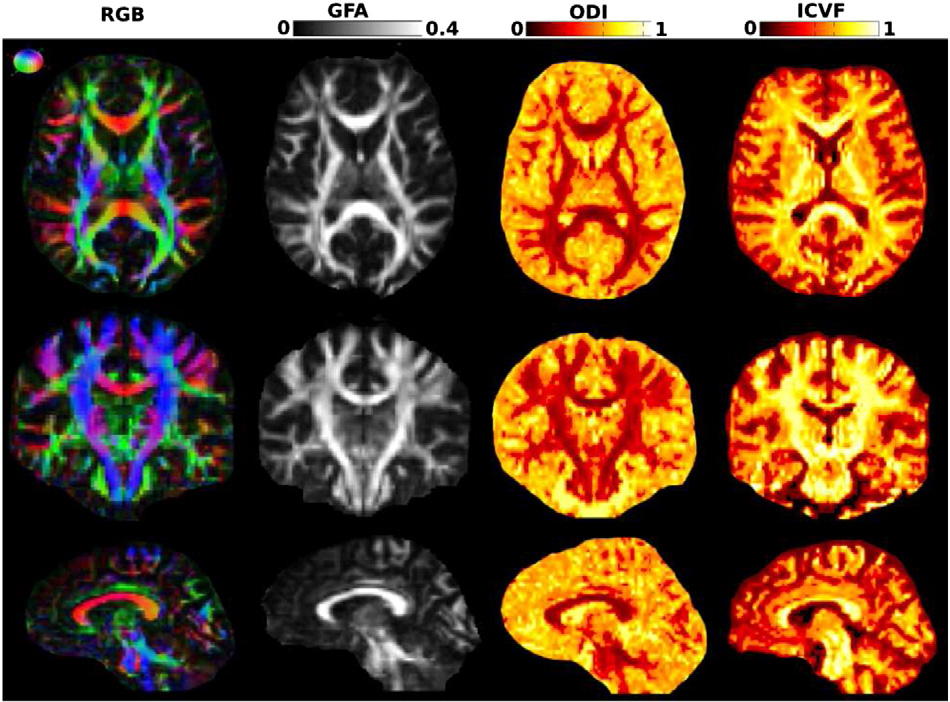
The color map of the RGB-encoded principal direction and the three different diffusion maps that are evaluated, GFA, ODI and ICVF of a single subject shown in three different planes, axial, coronal and sagittal.

**Fig. 2:**
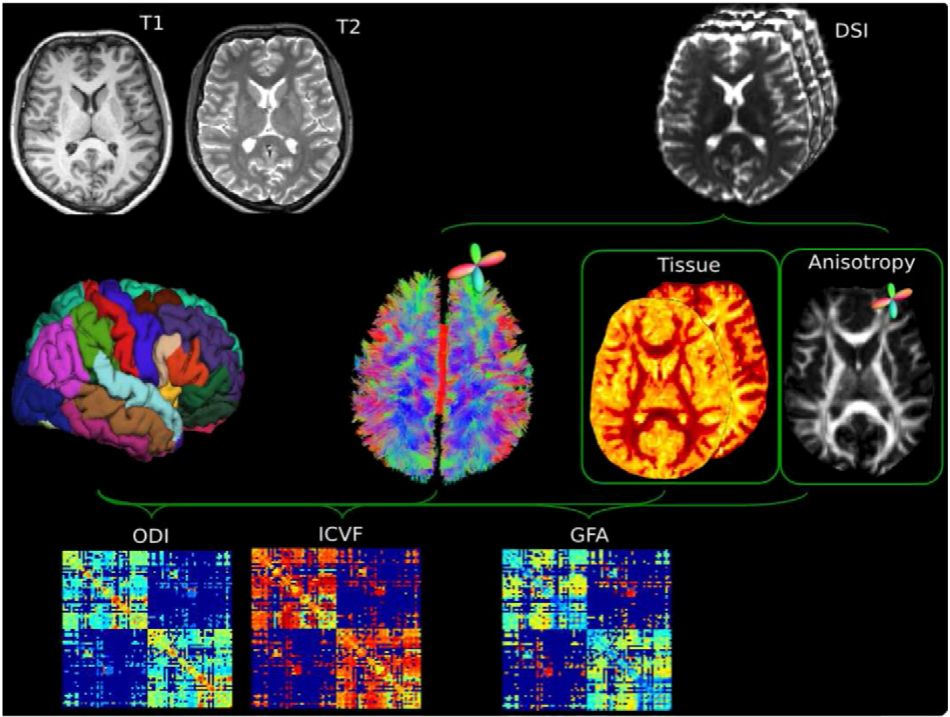
Connectome creation. The anatomical parcellation derived from the T1 images is registered to the diffusion space for the creation of the three connectivity matrices describing the GFA, ICVF and ODI along the tracts.

Three connectivity matrices (adjacency matrices) (Daducci et al., 2012) for each subject were built to represent the mean GFA, ICVF and ODI value values (Fig. 1) along the tracts between any pair of ROIs (Fig. 2).

These matrices can be seen as a network (also called graph) and they are mathematical descriptions of a system that is composed of interconnected elements, comprising a set of nodes and edges. The nodes (here, the brain regions) are the fundamental function units of the system. The edges are connections or links that relate the nodes to each other, these connections are weighted by the averaged GFA, ICVF or ODI. Furthermore, from these matrices several network measures can be computed describing the topology of the brain, a detailed description of these measures can be found in Rubinov and Sporns (2010).

All our graphs are weighted and the most fundamental graph measure is the **Strength (S)**. S is the sum of all edge weights (connections) of a node.

Nodes can be connected by single edges or indirectly by sequences of intermediate nodes and edges. We can express this with the **Characteristic Path Length (CPL)**, an indication of how well connected our network is. A fully disconnected graph would have a high CPL since all distances between the nodes are infinite. The global **Efficiency (E)** is a network measure that is directly related to the CPL, shortly it can be described as the inverse of CPL. Whenever CPL is high the E is accordingly low, therefore to reduce the number of computed measures we will only present the E in this study.

The Clustering Coefficient (CC) of an individual node (ROI) measures the density of connections between the nodes' neighbors. Densely interconnected neighbors form a cluster around the node, while sparsely interconnected neighbors do not. Measures of clustering highlight a particular aspect of the functional organization of the brain, its tendency to form segregated subsystems with specialized functional properties. While CPL and E describe the global interactions of the nodes in a graph, CC provides information about the network local community structure (clusters).

All the network measures mentioned above can be expressed in two ways: **Globally** and **Locally**. The global version is designed to give an overall indication of these measures in the brain network whereas the local measures look at the properties of individual regions. For the local changes, we restricted the analysis to E for consistency with previous studies in the field (Liu et al., 2014). Furthermore, the ROIs with the highest S (higher than one standard deviation above the mean) were classified as **Hubs** (Van den Heuvel and Sporns, 2014). These were computed to identify regions in the brain that are central to the neuronal communication since they are highly connected. We finally present in detail the local results obtained in both hippocampi as these structures are critical in TLE.

After computing these measures from each connectivity matrix belonging to every patient and control, we compared the global and local measures in RTLE vs controls and LTLE vs controls using a Wilcoxon rank sum test (p < 0.05) (Gibbons and Chakraborti, 2011; Hollander and Wolfe, 1999). At global level, we estimated correlations between network measures in patients and clinical variables (age of epilepsy onset, duration of disease) using Pearson correlation (p < 0.05). Concerning the local network measures, a Bonferroni correction was applied to correct for multiple comparison and False Detection Rate (FDR, p < 0.05) was applied to hub differences between groups.

## 3 Results

A chi-squared test showed no significant (p > 0.2) difference in gender between patients and controls. Similarly, the age was not significantly (p > 0.3) different using *t*-test.

### 3.1 Global measure

In RTLE, S (p = 0.0042), E (p = 0.0033) and CC (p = 0.0047) were all significantly lower than in controls when computed from the GFA weighting. Concerning the additional measures, S (p = 0.019), E (p = 0.029) and CC (p = 0.064, non-significant trend) were reduced in RTLE for the ICVF. For ODI, S and E were not statistically different but CC was reduced (p = 0.029). These results suggest that S and E alterations in RTLE were dominated by the ICVF contribution to the GFA, whereas CC alterations were driven by ODI.

In LTLE, there was no significant difference in any network measures based on GFA compared to controls. Regarding the network measures based on the ICVF along the tract, the CC (p = 0.0064) and E (p = 0.024) were significantly decreased while the S showed a strong but non-significant trend (p = 0.06). Measures based on ODI showed no trend or significant differences compared to controls. As in RTLE, the structural network alteration revealed by GFA analysis in LTLE appears to be driven largely by ICVF.

Therefore, RTLE and LTLE had a lower S, E and CC than controls when measures were based on GFA or ICVF along the tracts, whereas the contribution of ODI appeared less important.

**Fig. 3:**
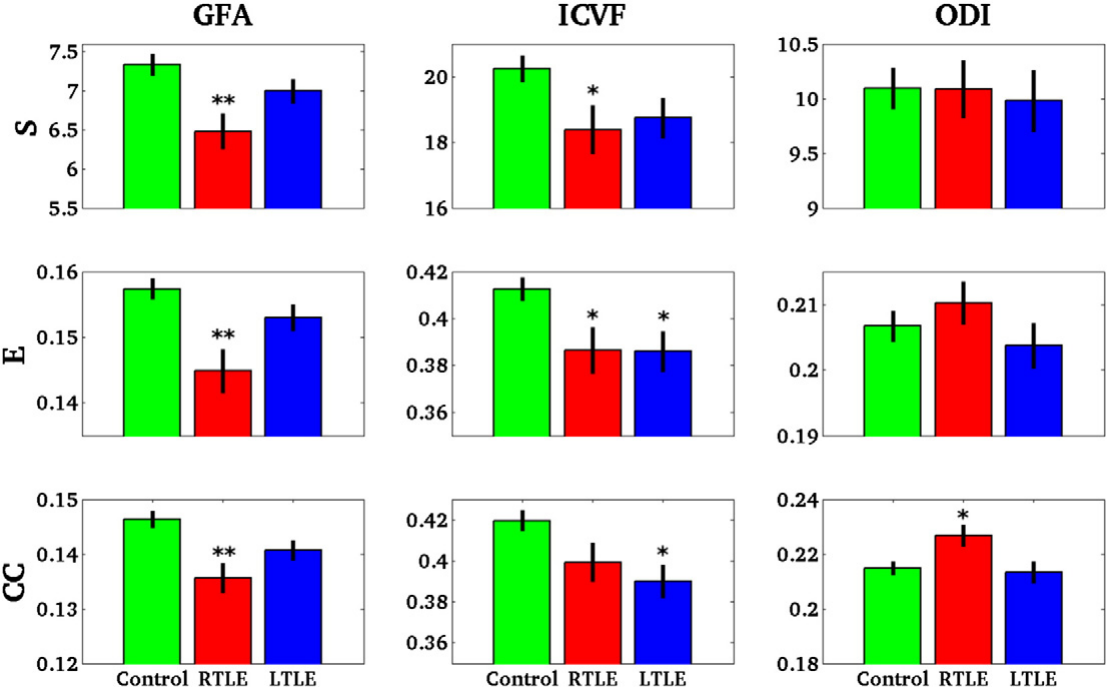
Global measures. S, E, CC based on GFA, ICVF and ODI along the tracts. *For p < 0.05 and **For p < 0.005.

Fig. 3 summarizes the results of E, S and CC based on all three scalar values (GFA, ICVF and ODI).

### 3.2 Clinical correlation

#### 3.2.1 RTLE

The RTLE showed a significant correlation for the CPL (almost significant for E) with the age of onset (p = 0.0314, r = 0.6202 and for E p = 0.0516, r = − 0.5727) when looking at the ODI along the tract.

#### 3.2.2 LTLE

In LTLE there was a significant correlation between S for GFA and duration of epilepsy (p = 0.044, r = 0.65) in parallel with a trend for both the ICVF and the ODI (p = 0.067, r = 0.6 and p = 0.059, r = 0.61). All other clinical correlations were not statistically significant.

### 3.3 Hubs

**Fig. 4:**
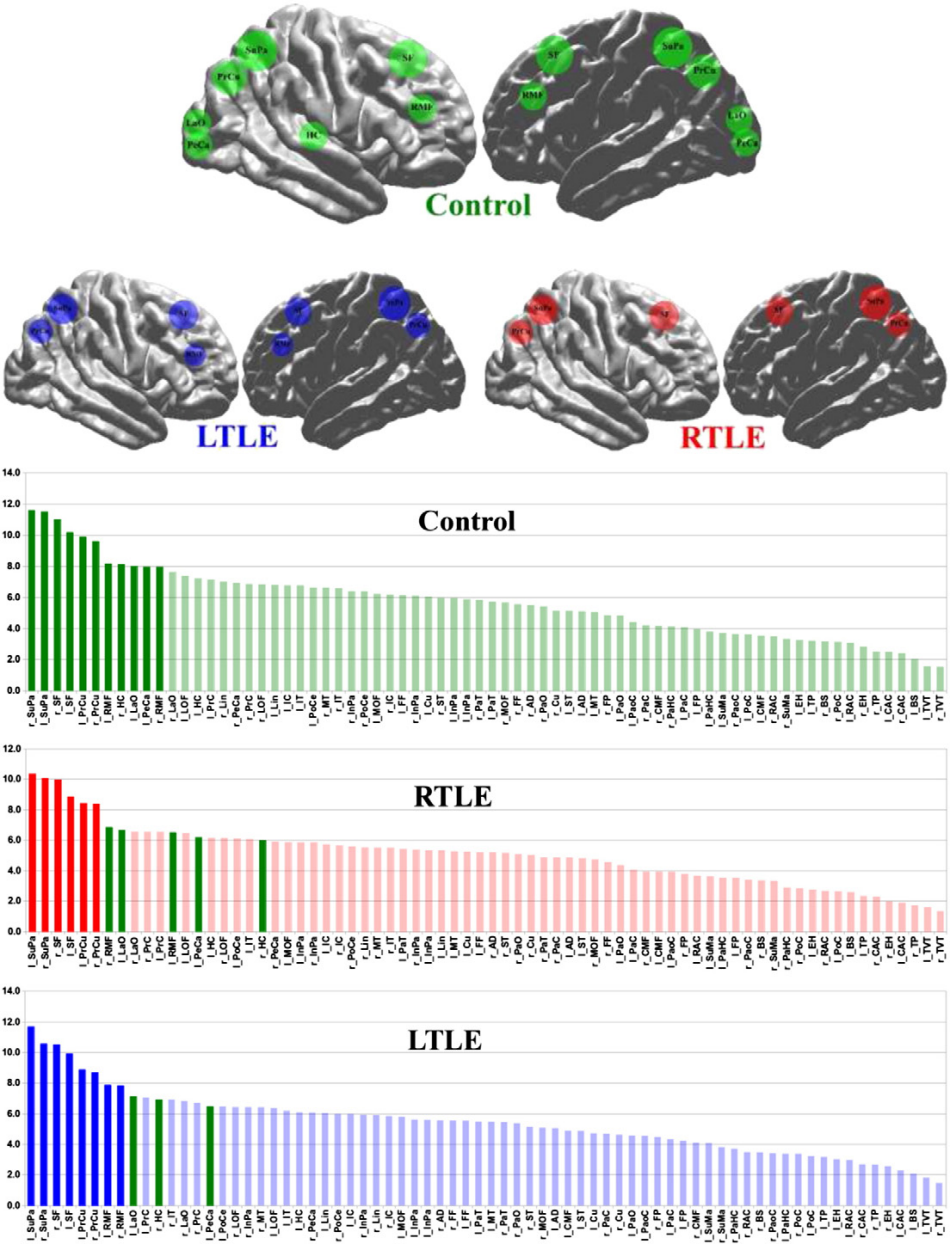
Top: Hubs shown on surfaces defined as regions with the highest strength (S > group mean + group SD) in the control, RTLE and LTLE. Down: Histograms showing the S distribution for the control, RTLE and LTLE groups. Dark colors indicate hubs. Green = hubs in control group, red = hubs in RTLE group and blue = hubs in the LTLE group.

For the GFA along the tracts 11 regions were classified as hubs in controls (Fig. 4). Among them are the bilateral superior parietal and frontal, precuneus, rostral middle frontal, left lateral occipital and left pericalcarine and the right hippocampus (left hippocampus not far from the threshold defined as mean + SD). In both RTLE and LTLE, the left lateral occipital and pericalcarine and right hippocampus were lost as hubs. In the RTLE, the bilateral rostral middle frontal regions were also lost.

Comparing hubs between groups, the right hippocampus was the most significant hub (p = 0.0001) in the RTLE followed by the left rostral middle- and superior frontal (p = 0.0007 and p = 0.0075 resp). The left hippocampus was also significant (p = 0.004). In the LTLE group however, only the left hippocampus was significant with a p-value of 0.0064.

### 3.4 Local measure

**Fig. 5:**
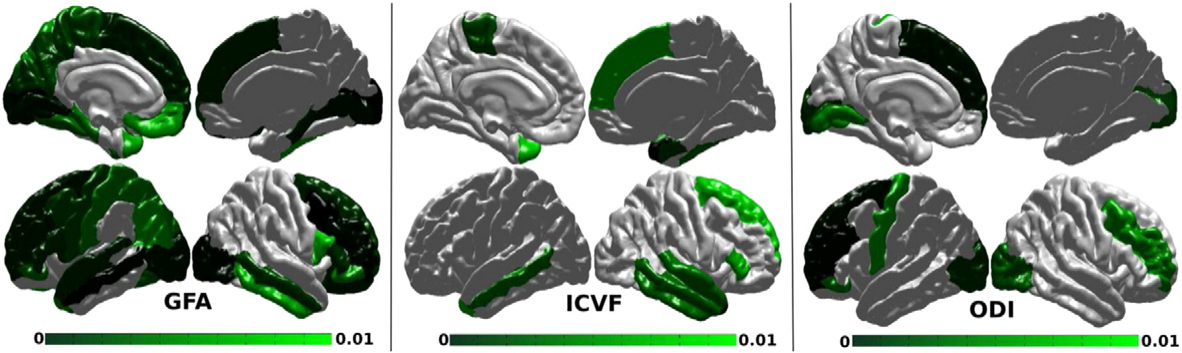
Local measures on RTLE vs control: E of nodes (ROIs) with p < 0.01.

Network nodes of the RTLE that contributed significantly to these alterations were located within as well as outside the temporal lobe in both hemispheres for all network measures (Fig. 5).

#### 3.4.1 RTLE

For the GFA-based local measures of E, both hippocampi as well as large portion of temporal and extratemporal cortex showed altered structural connectivity. The midtemporal gyrus, temporal pole, orbito-frontal dorso-lateral prefrontal and occipital regions were affected bilaterally. A higher number of altered regions were found on the contralateral (left) compared to the ipsilateral (right) hemisphere, notably in the pericentral area and the medial and lateral parietal regions. The E based on ICVF and ODI showed an interesting dissociation with an ICVF contributing rather to temporal lobe (and to a lesser extent to dorso-lateral prefrontal) changes, while ODI showing a predominant involvement of dorso-lateral prefrontal, pre-central and occipital regions.

All the regions with a p-value below 0.01 are shown in Fig. 5 for the E.

#### 3.4.2 LTLE

GFA-based decreases in local E were found predominantly in the left lateral temporal lobe, right occipital medial and bilateral orbito-frontal regions. More pronounced diffuse changes were found with ICVF-based measures. These alterations showed a similar but different pattern compared to RTLE: they involved the medial temporal, bilateral temporal polar and lateral temporal cortices, dorso-lateral prefrontal cortex and inferior/orbito-frontal cortex. Compared to RTLE, we notably found no alteration in the contralateral hippocampus and the contralateral precentral cortex. No significant changes appeared on ODI-based analysis.

**Fig. 6:**
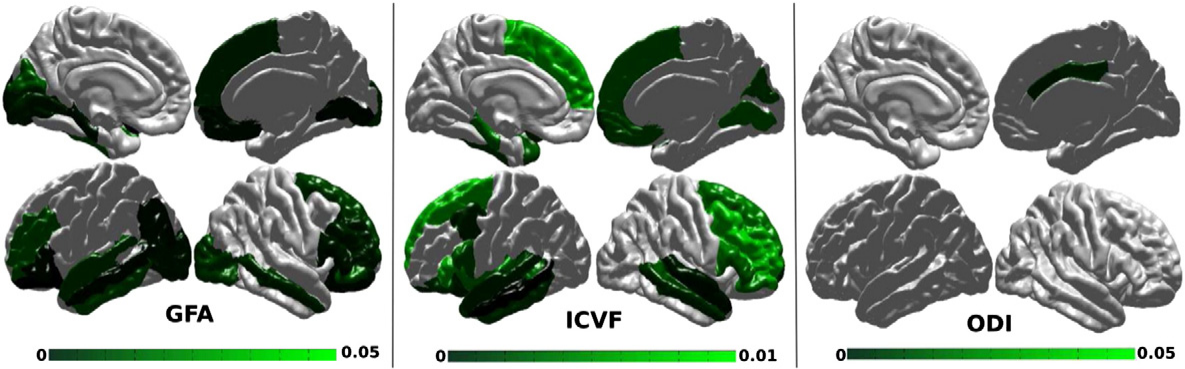
Local measures on LTLE vs control: E of nodes (ROIs) with p < 0.01 for the ICVF and p < 0.05 for the GFA and ODI.

Fig. 6 shows all the p-values below 0.01 for ICVF, but the threshold for the GFA and ODI was increased to 0.05 to visualize regions that are close to significance.

#### 3.4.3 Hippocampus

**Fig. 7:**
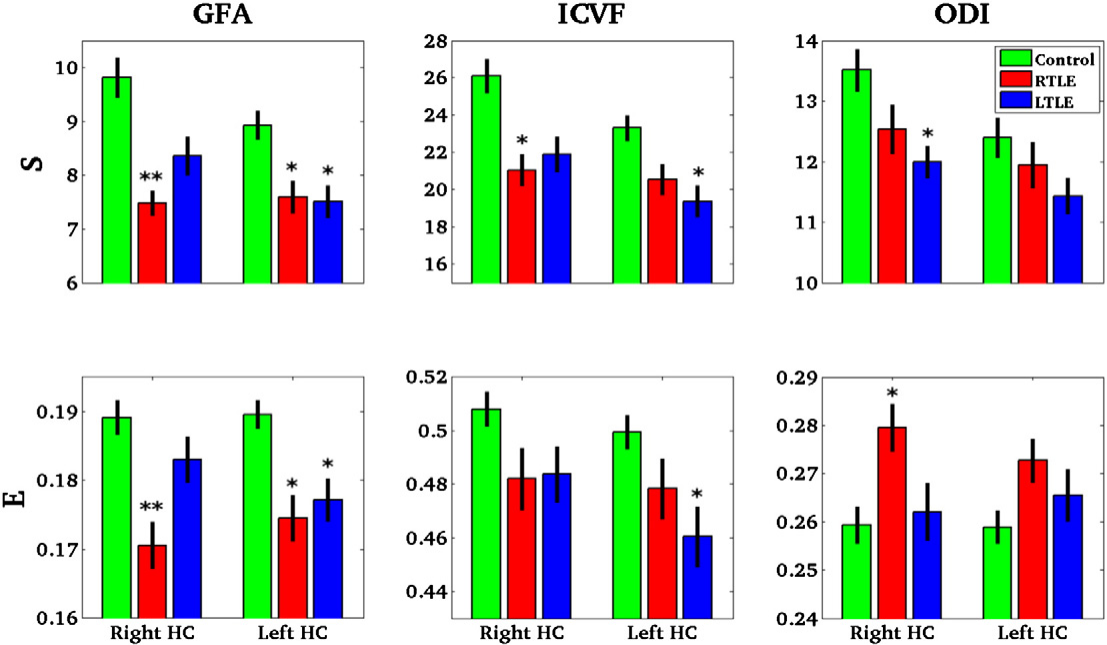
Mean and standard deviation of the three network measures, S and E computed from the three different connectivity matrices GFA, ICVF and ODI along the tracts within each of the three groups, control, RTLE and LTLE. ** = p < 0.0005 and * = p < 0.01.

In RTLE, S and E were affected in both hippocampi whereas in LTLE, only the ipsilateral (left) hippocampus was significantly affected for S and E. Regarding ICVF, the changes were significant only for the ipsilateral hippocampus (S for RTLE, S and E for LTLE). For the ODI-based measures, there was a trend for a more abnormal bilateral S in LTLE and bilateral E in RTLE. However, only some of the results were statistically significant (contralateral S for LTLE and ipsilateral E for RTLE) and the ODI-based E measure showed a bilateral increase RTLE that was discordant with GFA and ICVF measures (Fig. 7).

## 4 Discussion

Our study is the first application of tractography computed from DSI data, a high angular resolution diffusion technique, for investigating cortico-cortical structural connectivity changes in patients with epilepsy. The complex network alterations were described using graph analysis. We found bilateral, temporal and extratemporal connectivity alterations in LTLE and RTLE that are present at whole-brain level, hub level and regional level, notably in the hippocampus. LTLE and RTLE showed clearly different patterns of alterations. Moreover, a biophysical multi-compartment model, NODDI, allowed estimating the microstructural alterations underlying changes in GFA which is the measure commonly used in high angular resolution diffusion imaging (HARDI) studies.

### 4.1 Alterations in global connectivity measures in TLE

At a global whole-brain level and using GFA along the tracts, we found an altered network in RTLE with reduced E, S and CC. In LTLE, a similar trend was observed although statistical significance was not reached. Previous DTI studies concordantly showed global connectivity alterations in TLE using a similar approach with connectivity matrices and graph theory (Liu et al., 2014; DeSalvo et al., 2014; Bonilha et al., 2013) or whole-brain analysis of major white matter tracts (Focke et al., 2008; Voets et al., 2012). Other DTI studies focused on specific white matter tracts and concordantly showed widespread ipsilateral and contralateral changes in TLE (Gross et al., 2006; Concha et al., 2009; McDonald et al., 2008a; Yin et al., 2014; Lin et al., 2008).

Widespread structural brain abnormalities of the gray matter in TLE have been shown using volumetry (Bernasconi et al., 2003) voxel-based morphometry (Bonilha et al., 2004) cortical thickness (Lin et al., 2007; Bernhardt et al., 2008, 2010; McDonald et al., 2008b). Our cortical connectivity study assessed the white-matter dysconnectivity of cortical regions, rather than focusing uniquely on tracts, allowing to better integrate these multimodal findings.

Further, functional MRI (Liao et al., 2010) and EEG studies (Quraan et al., 2013) are in line with this demonstration of a widespread pathological network in TLE, confirming earlier electroclinical evidences (Lieb et al., 1991). Regarding the microstructural contributions to these global changes, neurite density, measured by ICVF, appeared to contribute more to these global changes than neurite orientation dispersion (ODI), with significant decreases for 2/3 network measures (S and E) in both RTLE and LTLE and strong trends in the other measures. This suggests that structural connectivity changes in TLE could be related to a loss of myelinated fibers rather than to altered geometric properties of the affected tracts.

### 4.2 Hub distribution in TLE

Zooming into the detailed architecture of structural connectivity, our study identified connectivity hubs including dorso-lateral and paramedian regions in controls and patients. These results were obtained by focusing on connectivity strength estimated using GFA, similarly to hub calculation in previous studies on clinical populations. Our results are concordant with the strongest hubs reported in healthy adult populations (Van den Heuvel and Sporns, 2014; Hagmann et al., 2008; Liu et al., 2014). In controls, we also found hub characteristics for the right hippocampus, while the left hippocampus had high strength but just missed the threshold for hub designation. Medial temporal hubs have been reported (Van den Heuvel and Sporns, 2014) while other studies did not detect them (Liu et al., 2014), probably in relation to the fact that connectivity studies based on DTI are limited by the relatively small size of temporal mesial structures. Weaker hubs in the temporal lateral regions (Van den Heuvel and Sporns, 2014) were not found in any of our groups, possibly in relation to small patient numbers. Interestingly, in both patient groups, bilateral medial temporal regions dropped in the hub hierarchy as compared to controls. In patients, we also found rearrangements in hub organization in the posterior cortex, concordant with previously reported shifts of hub localisation in LTLE (Liu et al., 2014). Moreover, changes in RTLE were also observed in dorsolateral frontal regions, concordant with the more disrupted network architecture in our RTLE group. A similar greater alteration of hub configuration in RTLE compared to LTLE was reported using cortical thickness measures in a larger population of TLE and controls (Bernhardt et al., 2011).

### 4.3 Regional alterations in TLE

At a regional level defined by atlas-based brain parcellation, we found altered connectivity of ipsilateral temporal, contralateral temporal as well as extratemporal regions in both TLE groups. These changes involved regions belonging to the limbic network, orbito-frontal regions, the lateral temporal cortex, dorsolateral prefrontal, precentral and occipital regions. Our regional maps in LTLE were similar to a recent connectivity study in LTLE which did not include RTLE patients (Liu et al., 2014). Focusing on the hippocampus, a key structure in local TLE networks, we found bilateral neurite density changes in RTLE whereas only the ipsilateral side was abnormal in LTLE. Orientation alterations were less consistent and therefore difficult to interpret. While early studies found that TLE was associated with bilateral changes in limbic networks (Concha et al., 2005), our results add up to the evidence that bilateral neocortical networks are also severely affected in TLE. The extratemporal regions showing differences in patients vs controls are concordant with changes in these regions reported with structural and functional studies. Strong anatomical and functional connections are known between occipital cortex and limbic temporal medial structures (Blume et al., 2005; Julia et al., 2008; McDonald et al., 2008a).

Altered connectivity of the precuneus has been reported by several DTI studies in TLE. In both our patient groups, the precuneus was conserved as a hub, although its connectivity was reduced at local level in RTLE but not in LTLE. Comparatively, Liu et al. (2014) reported the loss of hub characteristics and reduced connectivity of the precuneus in LTLE, while this region was not reportedly abnormal in another DTI study pooling RTLE and LTLE (Bonilha et al., 2013). Beyond differences between DSI and DTI, differences in brain parcellation and tractography strategies make comparisons difficult and further larger studies are warranted to investigate this issue.

Precentral cortical changes in TLE have been reported in DTI studies (Keller et al., 2014; Liu et al., 2014), cortical thickness studies (McDonald et al., 2008a) functional MRI (Voets et al., 2012) and ictal SPECT studies (Tae et al., 2005). Considering the microstructural estimates, earlier reports in focal epilepsy were limited to voxel-based analysis for lesion detection (Winston et al., 2014) or selective tracts (Lee et al., 2013). Using these metrics in our connectivity analysis, we found an interesting dissociation between temporal and extra-temporal lobe regions. The neurite density seemed more affected in the temporal regions whereas neurite orientation dispersion was more abnormal in extratemporal regions. One potential explanation for our findings could be that temporal lobe pathology is associated with cell and axonal loss at local (temporal) level that the remote (extra-temporal changes) is rather the result of disorganized fiber orientation and packing. Such alteration could be linked to altered tract plasticity with abnormal growth/pruning. The increased fragility of late vs early myelinating tracts in TLE could also fit with this hypothesis (Lee et al., 2013).

### 4.4 Different structural connectivity alterations in RTLE and LTLE

Our study is the first to systematically compare RTLE and LTLE structural networks at a whole brain scale and we observed different patterns of changes in RTLE vs LTLE. Our global, hub and local results, combined with investigation of microstructural signatures, revealed differences in the structural alterations underlying our patients with LTLE vs RTLE. RTLE showed a more extensive pattern of alterations affecting temporal and extratemporal structures with a more complex combination of microstructural changes. In LTLE, several regions had abnormal results based on ICVF without GFA changes (lateral and medial temporal as well as extra-temporal regions) suggesting that ICVF is a more sensitive marker of structural connectivity changes in LTLE than GFA. In RTLE, however, this effect seems much less present (lateral temporal only) and GFA changes showed the strongest abnormalities, resulting from a combination of ICVF and ODI alterations rather than only ICVF. Such asymmetries between LTLE and RTLE are in line with previous work in diffusion MRI (Voets et al., 2012) while another study reported more extensive changes in LTLE compared to RTLE (Ahmadi et al., 2009). In both studies, the repartition of hippocampal sclerosis vs non-lesional MRI in both groups was not detailed. More extensive and bilateral abnormalities in RTLE have also been highlighted by studies investigating other structural markers such as cortical thickness (Bernhardt et al., 2010) and cortical folding complexity (Voets et al., 2011). Likewise, connectivity studies based on functional imaging also support such asymmetry (Voets et al., 2012; Zhang et al., 2010).

The role of a different maturation speed between both hemispheres has been advanced as a potential explanation for these asymmetries (Voets et al., 2012). Such explanation is well in accordance with our results that global changes in RTLE correlated with age at disease onset while global changes in LTLE correlated with duration of the disease, although these demographic measures were not different between groups, highlighting different insults in the course of brain maturation and plasticity. Moreover, while the ICVF maps were similar in both groups, ODI showed changes in RTLE only and the differences between GFA maps in RTLE and LTLE suggest that the combination of ICVF and ODI changes could represent super additive changes.

We carefully investigated the effect of clinical factors as potentially relevant confounds. Patient gender, age, disease onset and disease duration were not different between both patient groups. This is concordant with DTI whole brain connectivity on LTLE that failed to find clinical or cognitive correlates to network metrics (Liu et al., 2014). In addition, Bonilha et al. (2013) found that post-operative seizure freedom in LTLE was associated with DTI-based lower connectivity between pairs of regions: ipsilateral medial and lateral temporal lobe, medial temporal and parietal lobe, contralateral temporal and parietal. In our study, subgroups of post-operatively seizure-free and non-seizure-free patients were too small for statistical analysis.

Distribution of etiologies was not statistically different between groups but showed a slightly higher proportion of patients with HS in the RTLE group (9/12, 75%) compared to the LTLE group (6/10, 60%). In previous studies, TLE with HS showed more profound and widespread structural changes than non-lesional cases but these differences seem to affect mostly the limbic circuitry while extra-limbic fiber bundles seemed equally altered in both situations. However, these studies have been performed on a user-specified selection of white matter tracts (Concha et al., 2009; Liu et al., 2012; Gong et al., 2008). In a number of other recent diffusion MRI studies, the exact pathology was not described or not confirmed and patients were pooled together (Voets et al., 2012; Bonilha et al., 2013; McDonald et al., 2008a; Ahmadi et al., 2009; Concha et al., 2009). Noteworthy, a number of functional and structural connectivity studies pooled patients with right-sided and left-sided pathology and reduced analysis to ipsilateral and contralateral changes. While such methodology might boost statistical power in small groups, the growing evidence of difference between RTLE and LTLE reported above highlights the limitations of such pooling, especially as groups are generally of different sizes so that the confounding effect of group mixing is difficult to compare between studies.

### 4.5 Methodological considerations

In this study, we acquired a DSI sequence with 128 diffusion directions and a maximum value of b that reached 6400 s/mm^2^ in 30 min (including DSI, field map, T1, T2). This allowed us also to obtain a higher angular resolution compared to a traditional DTI, but we were able to access the microstructural information in every voxel, due to the multiple b-values. However, DSI is not a unique method to derive both angular and radial profiles of the diffusion process. A recent review comparing different methods showed that other HARDI protocols can be used to obtain the same angular resolution in a shorter time (Daducci et al., 2014).

We used the NODDI model to describe the Intra-cellular Volume Fraction also referred to as the neurite density and neurite orientation dispersion. The NODDI was mainly developed to deal with multishell acquisition schemes where the highest b-value does not need exceed 2500 s/mm^2^. In our data higher b-values lead to a lower SNR which could potentially lead to a worse fitting when using NODDI. We have tested removing high b-value data in order to improve the fitting, but the effect on the results was minimal. Therefore, we chose to keep all the data as acquired and adapted the fitting to the SNR of our data.

High b-values lead to higher angular resolution but the drawback is the low SNR due to the weak gradient strength and the long echo time.

As a result of the long acquisition time, multiple averages were not acquired to improve the SNR. We focused more on winning in angular resolution (high b-values) and sacrificed some of the SNR, which has been shown to be more efficient (Jones, 2003).

Due to the low SNR for the highest b-values, an eddy current correction cannot be applied. Instead a twice refocusing spin-echo sequence with bipolar diffusion gradient encoding was used to minimize residual eddy-current sensitivity during acquisition (Reese et al., 2003). Furthermore, the intermediate step using the T2-weighted images remarkably improved the co-registration between the anatomical and diffusion space.

Considering that we have a high number of diffusion direction and high b-values to improve the crossing fiber issue in every voxel, we chose a simple tractography method as the deterministic streamline to compute a whole brain tractogram. Probabilistic methods are widely used on DTI data, including in epilepsy imaging studies, in order to improve the robustness and accuracy of tractography (Bonilha et al., 2013; Yogarajah et al., 2008). We have tested probabilistic tracking on our data but this led to a high number of false positive connections that could have been the consequence of the too rich information obtained from the ODF. We therefore opted for streamline tracking to aim at a lower number of true fibers rather than a too high number of false-positive fibers. Another aspect to consider is that the tractography itself is not a quantitative measure (Jbabdi and Johansen-Berg, 2011), therefore an indirect quantification of the connectivity is needed. The count of the number of streamlines connecting two regions in the brain demands some normalization that is hard to justify. Therefore, looking at scalar measures as the GFA, ICVF and ODI along the tracts takes us closer to the quantification of the true underlying neuronal structure.

Connectivity studies and graph analysis can be influenced by choices related to the construction of the networks. We kept the maximal information contained in the tractography analysis and used weighted graph as opposed to binarized thresholded analysis used in some other studies. It is very difficult to justify the set of threshold to binarize the connectivity matrix and to set any weight bigger than zero can lead to giving stronger importance to all small connection that might only be there due to noise.

It is difficult to investigate the connectivity of the deep limbic structures with a whole brain connectivity analysis but rather local and global effects of TLE on the cortex. These regions are more prone to error due to their small size and very easily effected by the partial volume and CSF for instance. Therefore, studies investigating FA changes on selected white matter bundles are difficult to strictly compare to our study. As mentioned above, we found concordant neocortical connectivity compared to previous DTI studies (Liu et al., 2014).

Finally, this study was an exploratory study on the use of diffusion anisotropy (GFA) and the microstructural tissue property (NODDI) in patients with epilepsy. The small size of our study population requires a larger study for confirming our findings and further investigating connectivity differences between RTLE and LTLE as well as the clinical correlates of the diffusion measures.

## 5 Conclusion

In this study, the network measures based on the structural connectivity estimated from the DSI data suggest that the network topology of the unilateral TLE is less efficient compared to the control group. We found both an intra-temporal and extratemporal alteration when considering a structural connectivity based on the anisotropy of the diffusion. To better understand these alterations, tissue microstructural parameters were included. The temporal changes were predominantly due to neurite density while the neurite orientation dispersion affected rather the extratemporal regions.

## Figures and Tables

**Table 1:** The clinical data of patients used in this study.

Patient	Gender	Age	Onset	Focus side	MRI	Intracranial EEG	ILAE OP outcome (follow-up in years)
1	F	32	9	R	HS	Yes	I (2 y)
2	F	42	20	R	HS		I (3 y)
3	F	37	25	R	HS		I (3 y)
4	F	36	12	R	HS	Foramen ovale	na
5	M	28	2	R	HS		I (3 y)
6	F	50	12	R	None	Yes	I (1 y)
7	M	38	2	R	HS		na
8	M	22	17	R	HS		I (3 y)
9	M	40	22	R	None	Yes	na
10	F	30	27	R	HS		na
11	M	36	20	R	HS		I (1 y)
12	F	20	5	R	HS		I (2 y)
13	F	15	4	L	HS	Yes	I (3 y)
14	M	18	8	L	None	Yes	I (3 y)
15	M	44	17	L	HS		I (1 y)
16	F	40	16	L	HS		na
17	F	43	37	L	HS		I (2 y)
18	F	25	13	L	None	Yes	II (1 y)
19	M	25	15	L	HS		na
20	F	48	36	L	None	Yes	III (6 m)
21	F	46	41	L	None		na
22	M	27	23	L	None	Yes	I (2 y)
